# Analyzing the Semantic Space of the Hippocratic Oath

**DOI:** 10.1515/med-2019-0079

**Published:** 2019-09-15

**Authors:** Přemysl B. Hanák, Kateřina Ivanová, Miroslav Chráska

**Affiliations:** 1 Department of Public Health, Faculty of Medicine and Dentistry, Palacký University Olomouc, Hněvotínská 3, 775 15 Olomouc, Czech Republic; 2 Department of Technical Education and Information Technology, Faculty of Education, Palacký University Olomouc, Žižkovo náměstí 5, 771 40 Olomouc, Czech Republic

**Keywords:** Hippocratic Oath, Evaluation of Hippocratic Oaths, Progressivity of the Hippocratic Oath, Medical practitioners

## Abstract

The Hippocratic Oath is the foundation for the moral ideals and goals of Western medicine. We aimed to develop a research tool to determine the perception among diverse groups of physicians and to determine the current perception of the Hippocratic Oath.

We used the semantic differential to map the connotative meaning of the Oath. We selected 34 research articles with abstracts from a literature search. The attributes (adjectives) used to describe the Oath were added to adjectives from a semi-structured questionnaire filled in at the Olomouc military hospital. We modified the factors and selected 8 scales by optimizing the semantic differential.

Overall, Czech physicians perceived the Oath highly positively based on the factor of evaluation. Negative and even neutral viewpoints were rare. The strongest factor– progressivity–revealed that the topicality of the Hippocratic Oath is very important to physicians. A statistically significant difference was found between female physicians and their male counterparts, with women rating the Oath’s progressivity higher than men, as well as perceiving the Oath generally more positively than men.

Our analysis confirmed the importance and success of continuing education. The standardized methodology can be used in medical education to analyze the semantic space of the Hippocratic Oath.

## 1 Introduction

The Hippocratic Oath is a “canonical text of medical ethics” [1]. As the apotheosis of strict ethical concepts in medicine, it is fundamental both to the patient-physician relationship and to maintaining high standards of professional morality. Over the centuries it has carried a powerful ethical message; it became pervasive throughout medicine with a remarkable endurance not only over time, but also among diverse cultures [2]. This demonstrates the close interrelationship between medical ethics and medicine itself as close, symbiotic disciplines.

The legacy of Hippocrates reveals the shared moral values that appear to be universal and timeless [3]. “Apart from the Bible, no document or author from antiquity has manifested the authority that Hippocrates of Kos and the Hippocratic Oath has had up to the twenty-first century” [4]. The Hippocratic Oath also alludes to the necessary congruence of biological, psychological, and social health, along with a systematic study of fundamental moral ideals and objectives [5]. It stems from the primary axiom of Hippocratic medicine, where the focus was not the disease itself but the patient as a person.

The tradition of the Oath has, nevertheless, been under continuous critical review. Critics point to its obsolescence mainly because of various modifications [6]. A further criticism has been its apparent failure to embody modern medical practice, including social and legal responsibility, research ethics, and the responsibilities in doctor-patient communication [7]. Some have even paraphrased Marx in calling it the “ethical opiate of medicine” or a “broken oath”, serving only as a shelter from hypocrisy [8]. In the role of the historical guarantee of quality in the medical profession, it has been tied to the Procrustean bed and its ethical principles have been stretched or lopped off depending on particular individual necessities [9].

With the objective of obtaining reliable conclusions, we focused primarily on gauging the opinions of medical professionals. The main aim of our study was to gauge the importance of the Hippocratic Oath among contemporary medical professionals. The design covered these research questions:

1. How do physicians perceive the Hippocratic Oath?
2. Are there differences in the perception of the Hippocratic Oath among different groups of physicians?
3. Can a standardized method be developed to determine the perception of the Hippocratic Oath among different groups of physicians in Western medicine?

## 2 Research design and methodology

Semantics explores the relationships between language expressions and the objects denoted by those expressions. To gather data, we used the socio-psychological semantic differential method, which focuses on the connotative meanings of words. Particular words evoke individual experiences, attitudes, emotions, opinions and knowledge, the influence of the environment, education, stereotypes, and prejudices, as well as the cultural and professional values of the respondents. Thus, the semantic differential can be used to compare the values or semantic dimensions (factors) of different terms that are formed as a result of their associations and psychological content.

Semantic space dimensions were primarily determined by factor analysis. The original Osgood Semantic Differential established the factors of evaluation, potency, and activity [10]. The factor of evaluation focuses on the ‘good or bad’ aspect of the term; the factor of potency on the strength of the term; and the factor of activity relates to the active or passive voice of the term.

According to Chráska [11], the assessment of the subjective meaning of terms using these three factors is too detailed. The extraction of three factors often led to a more ambiguous measurement, when on a single term, one scale excessively permeated into multiple factors. The third factor of activity was an especially fragile construct with the greatest risk of misinterpretation. A more detailed factor analysis found that only two factors contribute significantly to the distribution of values [12]: The factor of evaluation was taken from the original Osgood Semantic Differential, and the second factor was a combination of the Osgood factors of potency and activity, which Chráska called the factor of energy. This two-factor semantic differential–ATER (Attitudes Towards Educational Reality)–of Prof. Chráska formed the basis for our study.

If the Hippocratic Oath is perceived with different emphases and preferences in different groups, the semantic differential can be a useful tool to uncover the detailed structure of these differences within the community of medical professionals. This study is the first to apply the semantic differential to analyze the connotative meaning of the Hippocratic Oath: no similar study can be found in the Web of Science database.

The semantic differential measures the specific meanings of terms or attitudes through a number of rating scales, most often having over seven points. Each scale must be loaded with a single factor to ensure validity. The scale extremities are a pair of adjectives with opposite meaning (antonyms). Scales are presented in graphical form, and respondents record a numerical value corresponding to their rating of the relevant term. Joining these points yields a curve expressing the global and dimensional perception of the subject as well as the individual properties in the specific semantic space [13].

To construct the semantic differential scales, we analyzed abstracts of research articles and extracted only the Hippocratic Oath attributes mentioned therein to derive the adjectives and the corresponding antonyms. A total of 38 bipolar scales was derived by using all the attributes of the Hippocratic Oath found in the analysis of the abstracts.

There was no article in Czech among those selected. To expand the scope of the research tool into the Czech language, a questionnaire was distributed in the Olomouc military hospital in March 2017 with the aim of collecting other adjectives that characterize the Hippocratic Oath; the physicians were asked, “What four adjectives would you use to describe the Hippocratic Oath?” All 130 physicians in the hospital were given the questionnaire, 16 (12.3%) responded. All 26 adjectives found in the responses were used, and the same number of bipolar scales was created. Thus, the total number of bipolar scales created was 64. This relatively high number was retained as we expected a reduction based on the results of factor analysis. If a wider range of scales is used than is usual for a standardized research tool, there is always a proportional decrease in variance explained by the given factors [14]. We subsequently segregated the adjectives based on their presumptive association with the factors of evaluation and energy.

The semi-structured questionnaire designed in the study comprised two parts. The first included demographic queries required for sorting and statistical evaluation (gender, age, expertise, length of practice, etc.). The second consisted of a set of 64 attitude terms in the form of bipolar, seven-point scales. Respondents recorded their reaction according to which of the two adjectives was closest to their perception of the Hippocratic Oath. Attitude terms were both positive and negative. A positively tuned (standard) scale was assigned a value from 1 to 7 because the adjective in the positive sense was on the right side of the differential. In the case of negative adjectives, this was reversed (scales labelled R), from 7 to 1. Reversing scales prevents the stereotypical repetition of the rating process, which was achieved by randomly alternating reverse and standard scales. Further, we also alternated bipolar scales with different factor associations.

Before administration, the respondents listened to the full wording of the Hippocratic Oath in Czech, and they were free to refer to it at any time during the completion of the questionnaire. Furthermore, respondents were able to query any uncertainties about any item or method of completing the questionnaire. All queries were adequately resolved. The questionnaire was administered between September and November 2017 at the Faculty of Medicine of the Palacký University in Olomouc, as part of the specialized training of physicians; completion of the questionnaire never exceeded 30 minutes.

The questionnaire responses were checked and numbered. The data were recorded in numerical form in MS Excel and statistically evaluated using descriptive statistics in Statistica CZ software (version 13.4) by a statistician with a professional interest in the semantic differential. The hypotheses were tested at the α = 0.05 significance level. Statistical hypothesis testing was performed using a *t*-test as well as a one-way analysis of variance (ANOVA), and the χ^2^ was calculated.

During scale selection we ensured that we maintained the basic requirements of the semantic differential: relevance and representativeness. The requirement of relevance was met through the previous use of adjectives directly in the context of the Hippocratic Oath. The requirement for representativeness of the adjectives was verified by an exploratory factor analysis to confirm that the presumptive factors of evaluation and energy were present. Factor analysis made it possible to specify the basic common variables (factors) affecting the measurements with the particular term. Measurements with similar results showed a common factor, mainly due to the replacement of an extremely large set of data with a few identified factors.

## 3 Study cohort

In all, 140 physicians participated in the study: 51 men (36.43%) and 89 women (63.57%). The age range was 26 to 64 years. The average age was 32.65 y, and the median was 31 y. The average age among men was 33.47 y (median 32 y), and among women, 32.18 y (median 31 y).

The respondents included 12 fresh graduates (8.57%), 71 departmental physicians from the hospital (50.71%), 12 outpatient physicians (8.57%), and 40 department physicians who also performed outpatient duty (28.57%). The cohort also included 4 senior physicians (2.86%) and 1 superintendent (0,71%).

The shortest practice period was 6 months and the longest, 40 years. The average practice period was 6.45 y, (median 5 y). 16 respondents (11.43%) had practice experience of < 2 y, 63 respondents (45%) between 2.5 and 5 y, 47 respondents (33.57%) between 5.5 and 10 y, and 14 respondents (10%) had experience longer than 10 y. The number of individual specializations in the study cohort is shown in Table 1.

**Table 1:** Number and proportion of respondents by specialization

Specialization	Number	Percentage
General practice	19	13.57 %
Anesthesiology & Intensive care	16	11.43 %
Gynecology	13	9.29 %
Internal medicine	9	6.43 %
Pediatrics	9	6.43 %
General Surgery	7	5 %
Neurology	7	5 %
Oncology	6	4.29 %
Psychiatry	5	3.57 %
Orthopedics	4	2.86 %
Cardiology	4	2.86 %
Physical medicine & rehabilitation	4	2.86 %
Radiology	4	2.86 %
Hematology	3	2.14 %
Dentistry	3	2.14 %
Ophthalmology	3	2.14 %
Urology	2	1.43 %
Traumatology	2	1.43 %
Gastroenterology	2	1.43 %
Dermatovenerology	2	1.43 %
Orthopedics + Traumatology	1	0.71 %
Respiratory medicine	1	0.71 %
Microbiology	1	0.71 %
Emergency medicine	1	0.71 %
Internal medicine + gastroenterology	1	0.71 %
Internal medicine + Respiratory medicine	1	0.71 %
Nephrology	1	0.71 %
Dermatology	1	0.71 %
Ear, nose & throat	1	0.71 %
Pathology	1	0.71 %
Total (known specializations)	134	95.71%
Specializations non-mentioned	6	4.29%
Total number of respondents	140	100%

## 4 Results

The primary search for research articles was conducted in the Web of Science Core Collection with the search terms “Hippocrat* oath*/Title AND 2000-2015/Publication year”, for all spelling/language variants of the Hippocratic Oath. This time period was chosen to correspond with the intended study cohort of mostly young Czech physicians who began working in the field after 2000. In all, 117 entries were returned, 5 of which were duplicates or errors. The search revealed the frequency of the Hippocratic Oath over the given period, with 112 articles explicitly focusing on this subject over a period of 15 years. The fundamental criterion for selecting texts was the presence of an abstract, and 34 of the 112 included one.

Analysis of the abstracts showed an obvious segregation into three basic thematic categories:

1. Articles on the Topicality of Hippocratic Oath (n = 19)
2. Articles Applying an Oath in a Special or Ethnic Group (n = 8)
3. Articles dealing with the historical context of Hippocratic Oath (n=7)

A majority (56%) of the scientific texts with an abstract were about the topicality of the Oath. Most authors were

positive about its being the basic ethical standard for the medical profession, albeit with room for revision and modification; constructive criticism mainly concerned the form and not essential ethical principles. It was also viewed as a heuristic algorithm, a tool to discern the basic premise of ethical problems in medicine. Only one article was found that summarily rejected the Hippocratic Oath on principle.

Each text described the Hippocratic Oath in terms expressing the personal and qualitative perception of the author. Abstracts also used specific attributes. Table 2 summarizes these linguistic expressions.

**Table 2:** Linguistic perceptions of the Hippocratic Oath

Theme	Refinements (benefits) of the Oath	Revision (criticism) of the Oath
Topicality of the Oath (n = 19)	proto-occupation, text with intrinsic value, soul of professionalism, traditional value, public commit- ment, symbolic ritual, embodiment of medicine, starting point, medicinal doctrine, basic standard, moral authority, cornerstone, foundation of the medical profession, moral code, crux of medicine, classical text, philosophical remedy, guiding light, heuristics of medicine, guide for medicine, moral regulation, moral identity, symbol of medicine, transcendental text	new perspective, revision, text update, dialogue with text, choosing the optimal text, modern version of the Oath, new impetus, context of the words, Procrustean bed of medicine, text stabilization, value assessment, critical examination of the text, variant text conventions
Oath in a Special or Ethnic Group (n = 8)	living document, ethical template	text rationalization, pressure on medical neutrality, immunity, alternative values, relevance of the text, alternative approaches to the text
History of the Oath (n = 7)	ritual, basic principle, moral imperative, symbol of humanism, fascination for physicians, exemplary text, highly moral text, literary eloquence of the text, one of the best texts from antiquity	text evolution, different language, text version, constant text development, document of Pythagorean asceticism, new interpretation, text revitalization

38 bipolar scales were generated using inductive logic from the linguistic units, and they are listed (1 to 38) in Table 3. The remaining scales (39 to 64) were obtained from the questionnaire administered at the Military Hospital Olomouc.

**Table 3:** Scales with the respective loading factors

	Scales	Factor of evaluation	Factor of Energy	Reverse scale
1	original - innovative	0.063	0.412	
2	traditional - contemporary	0.116	0.543	
3	explicit - internalizing	0.220	0.312	
4	comprehensive - basic	0.285	0.343	R
5	expressive - commonplace	0.570	0.205	R
6	fundamental - intricate	0.545	0.013	R
7	equivocal - dogmatic	-0.250	0.130	R
8	static - dynamic	-0.013	0.645	
9	authoritative - permissive	-0.099	0.517	
10	strict - non-binding	-0.183	0.393	
11	amateur - professional	0.612	0.111	
12	natural - transcendent	-0.315	-0.255	
13	philosophical - material	0.160	-0.178	R
14	imperative - liberal	-0.084	0.466	
15	tolerant - restricting	-0.051	0.330	R
16	dead - viable	0.568	0.601	
17	identifiable - anonymous	0.262	0.113	R
18	mutable - stagnant	0.000	0.528	R
19	practical - symbolic	0.382	0.414	R
20	solitary - contextual	0.208	0.021	
21	cliched - varied	0.042	0.473	
22	aggressive - tolerant	0.191	0.605	
23	secular - ritual	0.059	0.121	R
24	unstable - stable	-0.517	0.051	R
25	fascinating - repulsive	0.592	0.298	R
26	schematic - stochastic	0.509	-0.030	R
27	inferior - superior	0.571	0.002	
28	convergent - divergent	0.273	0.117	R
29	cogent - epic	0.171	-0.194	
30	polythematic - monothematic	0.244	0.159	R
31	monologic - dialogical	0.060	0.533	
32	deteriorating - developing	0.396	0.482	
33	encouraging - demotivating	0.617	0.244	R
34	elemental - amorphous	0.659	-0.111	R
35	unconventional - conventional	-0.300	0.303	R
36	degenerative - evolutionary	0.427	0.596	
37	neutral - biased	-0.230	-0.373	
38	hedonistic - ascetic	-0.030	-0.325	
39	usable - unusable	0.618	0.510	R
40	ceremonial - practical	0.346	0.593	
41	non-binding - binding	0.561	-0.001	
42	contemporary - historical	0.275	0.761	R
43	true - false	0.668	0.254	R
44	simple - complex	-0.427	0.014	
45	universal - particular	0.321	0.265	R
46	factual - relative	0.481	0.320	R
47	necessary - unnecessary	0.707	0.355	R
48	long - short	0.183	0.090	
49	revered - dishonorable	0.293	0.172	R
50	unpretentious - demanding	-0.114	-0.176	
51	noble - undignified	0.623	0.092	R
52	idealistic - practical	0.281	0.496	
53	thoughtless - thoughtful	0.571	0.147	
54	obsolete - timeless	0.588	0.541	
55	reliable - unreliable	0.565	0.373	R
56	puritanical - liberated	0.088	0.600	
57	irresponsible - responsible	0.675	0.117	
58	doctrinaire - unheeded	0.227	0.248	R
59	meticulous - allegorical	0.484	0.472	
60	incomprehensible - understandable	0.407	0.004	R
61	imitation - original	0.592	0.079	
62	meritorious - indebted	0.386	-0.106	
63	venerated - facetious	0.724	0.039	R
64	original - innovative	0.368	0.188	R

**Figure 1:**
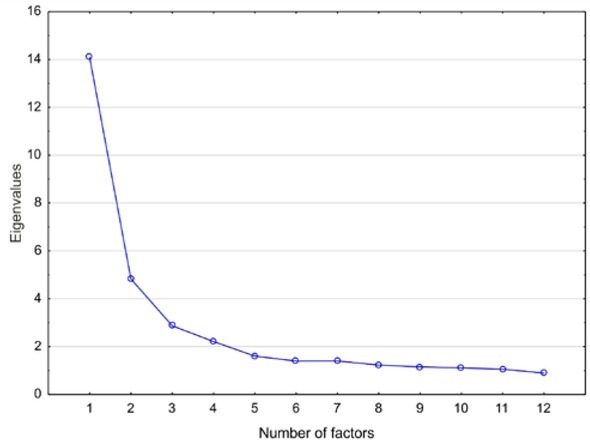
Plot of Eigenvalues

An exploratory factor analysis performed following statistical analysis of the bipolar scales obtained from the respondents showed that the individual scales did not always have the predicted factor structure. This meant that most scales were expressed insufficiently by two factors, or two factors significantly approached each other in one scale. Two factors explained only 29.10% of the variance and the residual correlations were significantly greater than 0 for most of the scales. Such values were thus unsuitable for evaluating the semantic differential, which to be useful, needs to explain more than 50% of the variance, with a residual correlation that is under 0.05 [11]. To approach such a variance value, at least 11 different factors would be necessary for one scale. Given this, the interpretation of the semantic differential would then become an extremely complex and subjective psychological construct, with virtually no real utility (see Fig. 1 for a scree plot).

Therefore, we reduced significantly the number of semantic differential scales to explain a greater variance with only two factors. This sort of “modulation” of the semantic differential by varying or reducing scales based on factor analysis is quite routinely applied. In our case, we applied maximum likelihood factor estimation and normalized varimax rotation. Based on the exploratory factor analysis, we selected 8 scales from the original 64 scales (containing bipolar adjectives) that fTaulfilled the conditions for a simple structure [15]. The selection criteria were: selecting the two strongest factors, comparable distribution of both standard and reverse scales, a saturation value of the predominant factor > 0.55 with the lowest possible saturation rate of the secondary factor. At the same time, these scales had to explain more than 50% of the variance, with a residual correlation lower than 0.05. Factor analysis in the last iteration confirmed the predicted two-factor structure with an excellent match for these scales.

The detailed structure of the two factors for the selected scales is shown in the following table 4, where values > 0.55 are marked in red.

**Table 4:** Factor loads

Scale	Factor of progressivity	Factor of evaluation	Reverse scale
traditional – topical	0.673	0.039	
static – dynamic	0.564	0.006	
ceremonial – practical	0.722	0.232	
contemporary – historical	0.832	0.159	R
principled – unethical	0.061	0.637	R
noble – undignified	0.085	0.654	R
irresponsible – responsible	0.149	0.709	
meritorious – dishonorable	0.074	0.798	R

When examining the selected scales initially assigned to the factor of energy, we found that this identification is not relevant. The scales expressed not only dynamism and energy, but also conservatism and traditionality, or innovation and modernity. Therefore, we decided to describe this aspect as a factor of progressivity. Factor analysis for the overall study cohort showed that this factor was even stronger than the factor of evaluation. In the semantic space of physicians, the progressivity aspect was more applicable for the term Hippocratic Oath than the evaluation component. Even more interesting was that it was the opposite when considering men only: the factor of evaluation for the Oath was stronger than the factor of progressivity.

The factor of progressivity explained 32.68%, and the factor of evaluation 18.29% of the variance; thus, the selected semantic differential scales described 50.97% of the scales’ variance. The factor match of the optimized two-factor semantic differential was excellent. The elements of the residual correlation matrix did not differ significantly from zero, as the χ^2^ test was not significant in the respondents [overall (p = 0.172); men (p = 0.162), women (p = 0.168)]. The reliability of the measurement decreased from the very high original value of α = 0.9 to α = 0.76, which is still acceptable.

**Figure 2:**
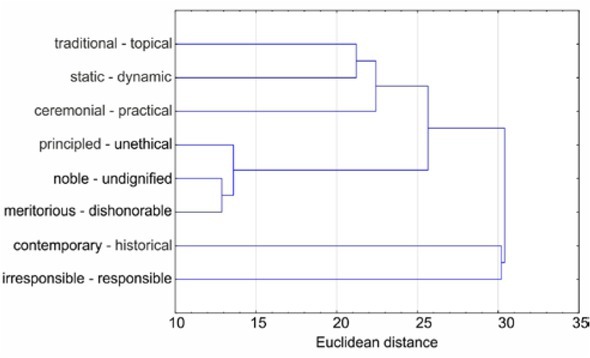
Dendrogram with 8 selected items for both men and women, measured by Euclidean distance

Figure 2 shows the 8 selected bipolar scales in the form of a dendrogram created using the hierarchical clustering method. It is a binary tree where each node represents one cluster. Horizontal sections of the dendrogram are deconvolutions from the clustering sequence. The vertical direction represents the distance between the individual clusters (decompositions).

So how did medical professionals perceive the Hippocratic Oath? Based on the overall measurement score, physicians perceived the Hippocratic Oath positively at

the level of both factors together; x = 4.597 (SD = 1.184). Men perceived the Oath almost neutrally x = 4.423 (SD = 1.399) and women positively x = 4.760 (SD = 1.445). Nevertheless, the range of values between 3.5 and 4.5 represented a neutral position. Values between 1 and 2.25 represented

**Figure 3:**
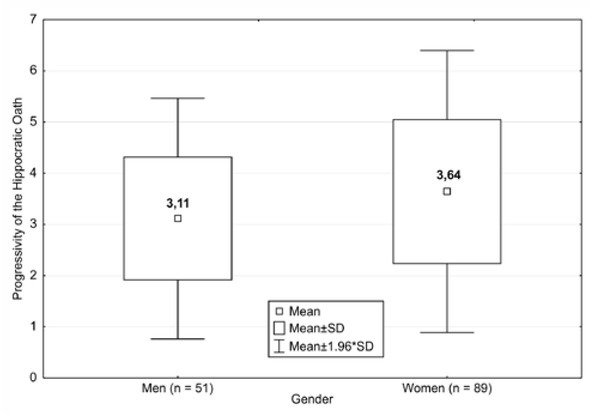
Factor of progressivity for the Hippocratic Oath

a strongly negative position, between 2.25 and 3.5 a moderately negative position, between 4.5 and 5.75 a moderately positive position and between 5.75 and 7 a highly positive position.

The overall factor of progressivity score for all respondents was x = 3.448 (SD = 1.354) and the overall factor of evaluation score for all respondents was x = 5.746 (SD = 1.014). We can therefore say that at the level of individual factors, physicians rated the Hippocratic Oath rather positively and considered it only moderately progressive; i.e., rather traditional, conservative, or ceremonial.

Did perception of the Hippocratic Oath vary among different groups of physicians? In particular, we focused only on statistically significant differences. As shown in Figure 3, the *t*-test found a statistically significant difference in the factor of progressivity levels between men and women (t = 2.25; p = 0.026).

The overall factor of progressivity score for men was x = 3.113 (SD = 1.96). Thus, men considered the Oath to be traditional, historical, and conservative. The overall factor of progressivity score for women was x = 3.640, a neutral value: Women considered the Oath neither too conservative nor unequivocally modern or practical, but a combination of both.

The overall factor of evaluation score for men was x = 5.603 (SD = 1.96), indicating a positive perception of the Hippocratic Oath. The overall factor of evaluation score for women was x = 5.829, indicating a highly positive perception of the Hippocratic Oath. However, this difference in perception between men and women was not statistically significant.

**Figure 4:**
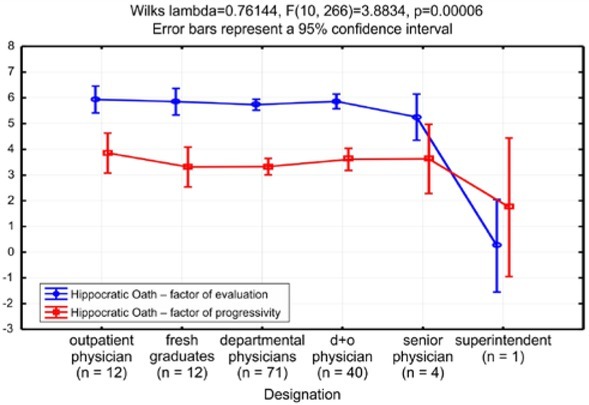
Line graph of factor distribution by designation

Other statistically significant differences were found between designations (Fig. 4).

The graph shows that the highest values of the factor of progressivity for the Hippocratic Oath were from outpatient physicians and senior physicians, and the lowest from the superintendent and fresh graduates. The highest factor of evaluation for the Hippocratic Oath was from outpatient physicians and department physicians who also perform outpatient duty. The lowest was from senior physicians and again the superintendent. The differences were statistically significant, with a Wilke’s lambda = 0.761 [F (10.266) = 3.883 and p <0.001].

Tables 5 and 6 show the standard and reverse scales that were given the highest values by the respondents. Note that the highest rating of reverse scales, as opposed to standard scales, is the most negative. However, it is necessary to keep in mind that these are data collected from all scales, i.e., before the semantic differential was optimized. The scales are therefore labeled with the initially anticipated factors of evaluation and energy.

**Table 5:** Standard scales with the highest rating

Scales	Number n	Mean	Minimum	Maximum	SD	Factor
authentic – imitation	139	5.90	1	7	1.25	energy
incomprehensible – understandable	139	5.83	1	7	1.16	evaluation
irresponsible – responsible	139	5.75	2	7	1.12	evaluation
amateur – professional	140	5.64	1	7	1.37	evaluation
insensitive – considerate	139	5.64	1	7	1.26	evaluation

**Table 6:** Reverse scales with the highest rating

Scales	Number n	Mean	Minimum	Maximum	SD	Factor
noble – undignified	139	5.89	1	7	1.12	evaluation
meritorious – dishonorable	139	5.83	2	7	1.23	evaluation
needed – unnecessary	139	5.78	2	7	1.39	evaluation
basic – complex	140	5.74	3	7	1.08	energy
principled – unethical	139	5.68	1	7	1.19	energy

**Figure 5:**
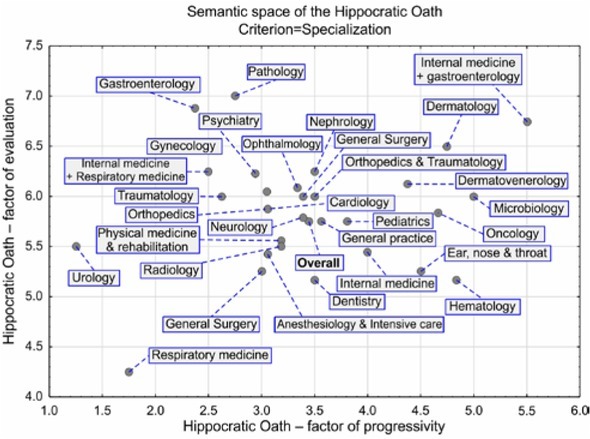
Semantic space of the Hippocratic Oath according to expertise

The final graphical output was a map of the semantic space of the Hippocratic Oath for the study cohort of Czech physicians (Fig. 5).

It is clear from the graph that the evaluation of the Hippocratic Oath and its progressivity was broadly similar for a large proportion of physicians with diverse specializations–low to neutral progressivity and positive to highly positive evaluation. Urologists scored the Oath the least progressive (very low), and physicians specializing in internal medicine and gastroenterology gave it the highest progressivity score. The lowest evaluation score was from the respiratory medicine specialist (neutral evaluation), and the most positive evaluation was from pathologists (maximum score of 7).

Can we propose a standardized methodology for validating the perception of the Hippocratic Oath in different groups of professionals in Western medicine? Our study demonstrated that we can determine the connotative meaning of the Hippocratic Oath in a particular cohort of physicians using a two-factor semantic differential. It must, however, be verified by factor analysis followed by a possible modification of factors and careful expert interpretation.

We selected 8 scales from the large initial number as useful for deriving a two-factor semantic differential. Using these scales in a study tool presupposes that valid measurement of the perception of the Hippocratic Oath is possible in more or less any cohort of physicians in the Western tradition. The proposed methodology enables future assessment of attitudes toward the Hippocratic Oath. Based on the results of these studies, it is also possible to modify the content and objectives of continuing education, especially in the field of medical ethics.

## 5 Discussion and conclusion

There were no significant differences between the assessment of the perception of the Hippocratic Oath in the study cohort of Czech physicians and results of the thematic analysis of abstracts of research articles on the Hippocratic Oath. This was further confirmed by a subsequent review of the full texts. Authors of research articles did not evaluate the Hippocratic Oath explicitly negatively, with one possible exception [16]. Czech physicians also rated the Oath positively–at the level of the factor of evaluation, the rating was highly positive. At the level of the factor of progressivity, the result was a low and somewhat neutral position. Therefore, they found it traditional, conservative, historical and ceremonial [17]. All the groups of physicians we identified evaluated the Hippocratic Oath positively, with women rating it highly positively. A neutral or negative evaluation was quite exceptional, with only one doctor rating it neutral and one as negative.

The factor of progressivity was stronger than the factor of evaluation in the overall physician population, and in particular, among female physicians. This is not typical for a two-factor semantic differential. However, this also corresponds with the thematic analysis of research texts, where the most frequent theme was topicality of the Hippocratic Oath. As a result, we observe that for Czech physicians the issue of progressivity, topicality, and the practical applicability of the Hippocratic Oath and its ethical message is very important for contemporary practice. Ptáček [18] reached a similar conclusion.

Differences in perception between younger and older physicians can be attributed in part to the relative paucity of experience of most respondents and their greater representation in the study cohort. Physicians with less than 5 years of experience (56.43% in total) rated the Hippocratic Oath better than their more experienced colleagues. In the Czech context of this study, physicians seem to focus mainly on rather pragmatic issues like limited financial resources, unnecessary paperwork, and strenuous treatment [19]. Also, worth mentioning is a certain ignorance and trivialization of the Hippocratic Oath and its relegation to a purely historical construct with a merely ceremonial purpose [20].

The study thus confirmed the characteristic multidimensionality of the semantic space, which can be analytically described only by carefully choosing the appropriate scales that are relevant to the subject under consideration. Using scales directly related to the concept being measured does not guarantee the necessary factor distribution. The semantic space can be divided into corresponding, unambiguously described parts only after scale optimization. The focus of the resulting scales then allows the factor of energy to be modified into the more applicable factor of progressivity. When using non-standardized scales, it is necessary to take this risk into account, as it cannot be unambiguously predicted [11].

Women physicians rated the Hippocratic Oath more positively and at the same time statistically perceived its progressivity as higher than men. This was observed also by Walton and Kerridge [21]. The higher evaluation is also affected by the greater proportion of women in the study cohort. To the contrary, Bourdieu [22] declared that excessive feminization has a debilitating effect on the prestige of any profession and, in general, also reduces interest in the medical profession.

Nutton [23] found that the relationship to the Hippocratic Oath is more positive in medical practitioners than among the leading lights on medical ethics, and we observed something similar in our study. The less positive rating for the Hippocratic Oath among senior physicians is likely linked to their bearing the responsibility for rapid and efficient performance of the healthcare unit. In this role, however, they ought to function as professional guarantors, positively influencing the ethical practices and behavior of their subordinates [24].

That we did not find commonalities in the ratings of the Oath in closely-related specialties was probably due to the limited number of physicians in the cohort. A broader study will very likely uncover common features.

Based on the highest score on the standard scales, we find that the cohort of physicians perceived the Hippocratic Oath as highly original, highly understandable and highly responsible. On the other hand, based on the reverse scale scores, respondents perceived the Hippocratic Oath as undignified, dishonorable, and even unnecessary. A similar ambiguity in the perception of the Hippocratic Oath is highlighted by Antoniou et al. [25]. This is generally understood to be the result of the ambivalence of young medical professionals towards conservatism and traditionality. Bombeke et al. [24] also point out the rise in critical attitudes towards conservative values and the crisis of empathy in the current medical generation.

This diversity is probably due mainly to the different values among individuals and their different ethical background. According to Casella et al., [26] the exploration and establishment of a personal ethical foundation is not just a matter of status or education. It also depends on the personality, experience, and background of the individual as well as on comparison with the experience of others.

The Hippocratic Oath can play an important role here as well, by exemplifying the basic professional ethical principles and helping strengthen them. The conscientiousness of the doctor, reinforced by a relevant ethical code, is by far the most important factor in ensuring good medical practice and patient safety [27].

This individualized and rather ambivalent approach to classical principles, here represented by the Hippocratic Oath, can be regarded as an affirmation of the modern principle of autonomy, not only of the patient but also on the part of the doctor. This is at the expense of the original ethical principles, including benevolence and, in particular, the principle of doing no harm. A study of the ethical dilemmas in contemporary medicine [19] came to the same conclusions.

Along with relativization of traditional values comes a difficulty in the establishment of generally applicable objectives for continuing education in medical ethics. Education is the primary introduction to professional ethical identity, including the Hippocratic Oath [20, 28, 29]. The highly positive evaluation of the Hippocratic Oath among the youngest generation of physicians confirms the importance of the educational process and validates its success, as can be seen from our conclusions. As Revill & Dando [30] have pointed out, we need to build upon this validation.

Our study shows that the Hippocratic Oath continues to resonate strongly with the medical profession. Given its profound influence on the history of medicine and on cultural awareness generally, it is clear that it must be conserved as something precious with undiminished value. Our analysis of research articles also revealed a profound admiration for the humanistic universalism of the Oath even in non-medical fields where the focus was more on its philosophical message [31, 32, 33]. The ripples of its professional wisdom affect even fields distantly related to medicine, while at the same time reminding us that in medicine, philosophy has the same weight and stature as empiricism. In this light, Hippocratic medicine can be seen as the foundation of a new humanism that recreates a significant and secure ethical objective in a globalizing world [34].

We limited our study to the 2000-2015 period. The articles thus mainly describe recent attitudes to the Hippocratic Oath. The primary analysis was restricted to abstracts of research articles. A follow-up research dissertation analyzed the full text of all articles fulfilling the described criteria, as well as those published till the end of 2018. This review of full texts fully confirmed the results of the initial thematic analysis based on abstracts alone.

In some cases, the number of respondents in individual specializations or designations was very low. Nevertheless, for analyses of the semantic differential, the semantic space of each individual is as important as that of the whole group. For a more precise description of the Hippocratic Oath and its position within the semantic spaces of the study cohort, it would be appropriate to analyze other associated concepts. However, such an analysis is beyond the scope of the presented study, focusing mainly on the Hippocratic Oath.

All respondents were fully informed that the collected data are anonymous and will be used for research purposes only.

## References

[1] Bazylevych MY (2015). Ukrainian Physicians Reinterpret the Hippocratic Oath: Significance of Remuneration and Class in Bioethics. Human Organization.

[2] Davey LM (2001). The Oath of Hippocrates: An Historical Review. Neurosurgery.

[3] Baltussen H. (2015). “Hippocratic” Oaths? A Cross-Cultural Exploration of Medical Ethics in the Ancient World. Frontiers of Ancient Science: Essays in Honor of Heinrich von Staden.

[4] Nutton V (2004). Ancient medicine.

[5] Kumar A (2010). Hippocratic Oath, 21st Century. Indian Journal of Surgery.

[6] Jotterand F (2005). The Hippocratic Oath and Contemporary Medicine: Dialectic Between Past Ideals and Present Reality?. The Journal of Medicine and Philosophy.

[7] Loewy EH (2007). Oaths for physicians-necessary protection or elaborate hoax?. MedGenMed.

[8] Pi D (2012). Hiding Behind the Hippocratic Oath. Journal of General Internal Medicine.

[9] Ban D (2012). An Application of the Hippocratic Oath to Modern Medical Ethics. Korean Journal of Philosophy of Medicine.

[10] Osgood CE, Suci GJ, Tannenbaum PH (1957). The measurement of meaning.

[11] Chráska M. (2014). The application of a factor analysis to verify the factor structure of modified semantic differentials for measuring students’ attitudes. Proceedings of the SGEM2014 Conference on Psychology and Psychiatry, Sociology and Healthcare Education. Albena, Bulgaria.

[12] Chráska M, Chrásková M (2016). Semantic Differential and its Risks in the Measurement of Students’ Attitudes. Procedia - Social and Behavioral Sciences.

[13] Kubiatko M (2016). Semantic Differential as One of the Possibilities of Investigating Lower Secondary School Pupils’ Attitudes toward Chemistry. Scientia in educatione.

[14] Chvál M, Vašťatková J (2010). Using the semantic differential in school self-evaluation (in Czech). Orbis Scholae.

[15] Thurstone LL (1947). Multiple factor analysis.

[16] Colvin BT (2003). Why we do not need a Hippocratic Oath. Medical Education.

[17] Hanák P, Ivanová K (2018). Case study from the perspective of Hippocratic medicine (in Czech). Pediatrie pro praxi.

[18] Ptáček R, Bartůněk P, Mach J (2017). Informed consent: ethical, legal, psychological and clinical aspects (in Czech).

[19] Hanák P, Ivanová K (2018). Revitalization of the Hippocratic Oath? (in Czech). Časopis zdravotnického práva a bioetiky.

[20] Hanák P, Ivanová K (2019). What remains of the Hippocratic Oath in current medical covenants? (in Czech). Praktický lékař.

[21] Walton M, Kerridge I (2014). Do no harm: is it time to rethink the Hippocratic Oath?. Medical Education.

[22] Bourdieu P (2000). La domination masculine (Czech translation).

[23] Nutton V (1997). Hippocratic medicine and modern morality. Médecine et Morale dans l‘Antiquité, Entretiens sur l‘Antiquité Classique.

[24] Bombeke K, Winter BD, Royen PV (2014). Attitude erosion in medical students: dwarf or devil, fact or fable?. Medical Education.

[25] Antoniou SA, Antoniou GA, Granderath FA, Mavroforou A, Giannoukas AD, Antoniou AI (2010). Reflections of the Hippocratic Oath in Modern Medicine. World Journal of Surgery.

[26] Casella C, Capasso E, Terracciano L, Delbon P, Fedeli P, Salzano FA (2018). Ethical and legal issues in gestational surrogacy. Open Medicine.

[27] Catto G (2013). The Hippocratic Oath: back to the future?. Medical Education.

[28] Pellegrino ED (2001). The Internal Morality of Clinical Medicine: A Paradigm for the Ethics of the Helping and Healing Professions. The Journal of Medicine and Philosophy.

[29] Holmboe E, Bernabeo E (2013). The ‘special obligations’ of the modern Hippocratic Oath for 21st century medicine. Medical Education.

[30] Revill J, Dando MR (2006). A Hippocratic Oath for life scientists: A Hippocratic-style oath in the life sciences could help to educate researchers about the dangers of dual-use research. EMBO reports.

[31] Matteucci R, Gosso G, Peppoloni S, Piacente S, Wasowski J (2012). A Hippocratic Oath for geologists?. Annals of Geophysics.

[32] Diokno AC (2010). Editorial comment: Hippocratic Oath and plagiarism. International Urology and Nephrology.

[33] Ho Y-C (2015). A modest proposal for a new beginning: a Hippocratic Oath for S&T workers. National Science Review.

[34] Mikołajewska E, Mikołajewski D (2013). Ethical considerations in the use of brain-computer interfaces. Central European Journal of Medicine.

